# Aortic root in competitive sports: eyes on the older athlete

**DOI:** 10.1136/heartjnl-2019-314759

**Published:** 2019-04-10

**Authors:** Harald T Jorstad, Maarten Groenink

**Affiliations:** Departments of Cardiology and Radiology, Amsterdam University Medical Centre, location AMC, Amsterdam, NH, The Netherlands

**Keywords:** sports cardiology, aorta, screening, aortic aneurysm

Individuals with a suspicion of aortic disease are discouraged to take part in most competitive sports.^1^ In the more prevalent and syndromic forms, such as Marfan syndrome (prevalence 1: 5000), a sports examination with echocardiography may reveal this condition, with great consequences for the fate (and especially career) of a young athlete. From the point of view of the most recent guidelines,^1 2^ the prevention of aortic dissection is emphasised as the main task of clinicians. Cut-off values and z-scores are supplied to identify those that could be at risk for a pathophysiological process leading to cardiovascular morbidity and mortality. At the same time, sports physicians are often struggling with the cases that may be less clear, where slight aortic dilatation may be considered physiological. While the initial steps are evident—thoroughly investigate if there are any signs of underlying diseases such as Marfan syndrome or related conditions—in individuals with a mild dilatation of the aortic root without any other signs of an underlying disease, the next steps to be taken can be less clear. The increased availability of genetic panels to detect inborn predispositions for a wide range of aortic disorders may tempt sports physicians to reach for this diagnostic modality. However, uncertain pathogenicity and extreme variable penetrance of found mutations may just further obscure the situation.

**Figure 1:**
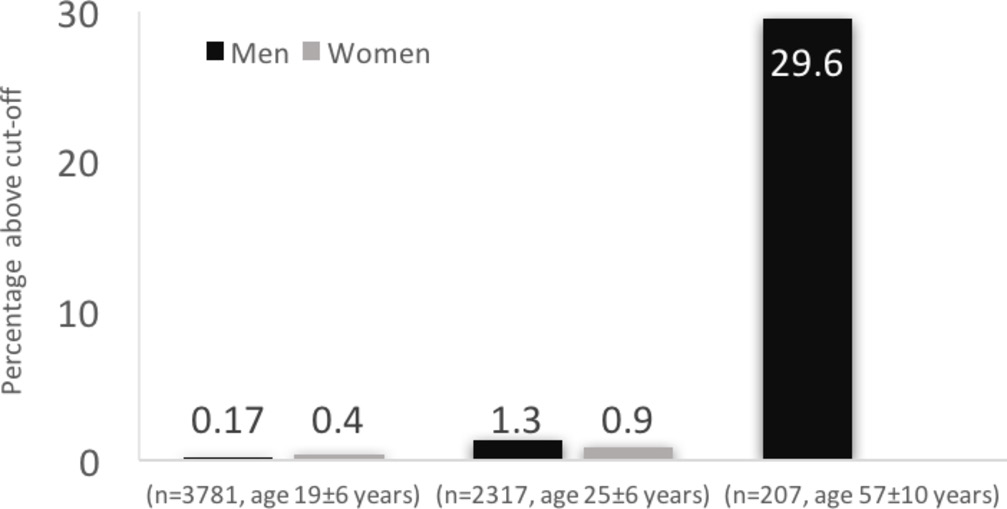
Prevalence of aortic dilatation in athletes in three age categories. Legend: prevalence of aortic dilatation above predefined cut-offs according to study. Gati *et al* 3 (n=3781, age 19±6 years): aortic root 99th percentile: men: 40 mm, women: 38 mm. Pelliccia *et al* (n=2317, age 25±6 years): aortic root 99th percentile: men: 40 mm, women: 34 mm. Gentry *et al* 7 (n=207, age 57±10: years): ascending aorta 40 mm in men.

Current data from different groups of athletes suggest that training on its own has only a limited impact on physiological aortic root remodelling (figure 1).^3 4^ This impression is strongly reinforced by the results presented by Gati *et al* 5 in this issue of *Heart*, who echocardiographically investigated aortic root diameters in 3781 athletes aged 19±5.9 years, in whom hereditary thoracic aortic disease was definitely ruled out. These aortic dimensions were similar to a control population of 806 individuals when corrected for BSA. The 99th percentile in the athlete population was 40 mm in men and 38 mm in women, and only five men (0.17%) and six women (0.4%) had an aortic root diameter above the 99th percentile. After a mean follow-up of 5±1.5 years, none of these athletes showed a significant increase in their aortic root dimensions, and there were no aortic events in the entire athlete population. In short, a severely dilated aortic root without an underlying disease is seldom found in young athletes, and in athletes with mild enlargement, there seems to be no clinically important progression of the dilatation, despite of ongoing participation in sports over a period of approximately 5 years. The authors therefore suggest that ‘there is scope for being more liberal in athletes with a slightly enlarged aortic diameter in the future although annuals assessments are recommended’.

A number of important factors should be kept in mind when assessing and performing follow-up of the athlete’s aortic root. First, when measuring the aortic root using two-dimensional echocardiography in the parasternal long axis, the assumption is made that this is the real representation of the sinus of valsalva. However, a cross-sectional echo plane could well inadvertently intersect the vessel obliquely or not intersect the midline of the vessel, leading to an underestimation or overestimation of the diameter.^6^ Considering that 2 mm or even 1 mm growth in aortic root diameter should be viewed as highly suspicious in athletes, as suggested by Gati *et al*, reproducibility of findings is of importance when performing follow-up.

Second, the epidemiology of aortic dilatation teaches us that most dilatation takes place later in life. Gentry *et al* have previously shown that the prevalence of dilatation of the ascending aorta as measured by computer tomography among 207 middle-aged (57±10 years), male, former American-style football players was almost 30% using >40 mm as a cut-off and 9% using ≥45 mm as a cut-off (figure 1).^7^ This is in sharp contrast to the numbers reported in young athletes. Is this primarily driven by the changes in lifestyle and progression of classical cardiovascular risk factors such as hypertension after cessation of a professional sports career, or are these changes secondary to sports-related factors, such as repetitive injury to an aorta with a propensity to dilate or to the demands of high-dynamic sports performance? The medium-term follow-up performed in the study by Gati *et al* serves to reassure us for the early parts of an athlete’s career, but studies are urgently needed to investigate the complex interplay between detraining and cessation of a professional sports career, the associated changes in lifestyle and ageing.

Third, while sudden cardiac death due to aortic diseases is rare in the elite athlete,^8^ the question remains if the enlarged aorta of an athlete carries the same propensity for dissection as in the general population, both in youth and later in life. While a liberal approach is probably warranted in the young athlete with mild aortic root dilatation, clinicians should remain vigilant in athletes at the end of their sports career and in later life. Prospective, longitudinal data are needed across a wide variety of sports and ages for further understanding the interaction between health and sports and to identify those individuals in whom too much of a good thing, in this case sports, might actually be a bad thing.
